# Promoting Patient Safety: Exploring Device-Associated Healthcare Infections and Antimicrobial Susceptibility Pattern in a Multidisciplinary Intensive Care Units

**DOI:** 10.7759/cureus.50232

**Published:** 2023-12-09

**Authors:** Neeta Gade, Ranga Burri, Akkilagunta Sujiv, Meena Mishra, B.E. Pradeep, Harish Debaje, Tejswini Sable, Amandeep Kaur

**Affiliations:** 1 Microbiology, All India Institute of Medical Sciences, Nagpur, Nagpur, IND; 2 Public Health, University of Hyderabad, Hyderabad, IND; 3 Community Medicine, All India Institute of Medical Sciences, Nagpur, Nagpur, IND; 4 Biosciences, Sri Satya Sai Institute of Higher Learning, Puttaparthi, IND; 5 Hospital Infection Control, All India Institute of Medical Sciences, Nagpur, Nagpur, IND; 6 Nursing, All India Institute of Medical Sciences, Nagpur, Nagpur, IND

**Keywords:** healthcare associated infections (hai), device associated healthcare infections, antimicrobial resistance, intensive care unit, infection control

## Abstract

Introduction: Device-associated healthcare infections are among the prevailing threats to patient safety worldwide. They constitute the third most common adverse event during healthcare delivery, resulting in heightened morbidity, mortality, and healthcare costs. Patients in intensive care units (ICUs) are at increased risk for device-associated healthcare infections. Focused active surveillance is a crucial measure for assessing the prevalence of healthcare-associated infections and controlling the transmission of pathogens, ultimately contributing to the establishment of quality outcome indicators. This study aimed to investigate and establish the baseline rates of healthcare-associated infections associated with medical devices in adult multidisciplinary ICUs within a tertiary care institute.

Material and methods: This hospital-based prospective observational study was conducted in two adult ICUs of a tertiary care institute in Central India over nine months. Targeted active surveillance for three device-associated health care infections namely central line-associated bloodstream infection (CLABSI), catheter-associated urinary tract infection (CAUTI), and ventilator-associated event (VAE) was conducted as per the Center for Disease Control (CDC)/National Healthcare Safety Network (NHSN) 2016 surveillance definitions and criteria. Pathogens associated with device-associated healthcare infections were identified and their antimicrobial susceptibility profile was studied.

Results: During the study period, a total of 5,773 patient days were investigated. Of 1,270 patients, 28 episodes of device-associated healthcare infections were detected in 26 patients, this suggests a collective occurrence of five device-associated healthcare infections for every 1,000 patient days in the ICUs. The device utilization ratios of the central line, mechanical ventilator, and urinary catheters were 0.33, 0.27, and 0.68, respectively. VAE, CLABSI, and CAUTI rates were 8.92, 5.68, and 0.76 per 1,000 device days, respectively. The most common pathogen isolated from device-associated healthcare infections was *Klebsiella pneumoniae* (39%) followed by *Acinetobacter baumanii* (22%). The majority (82.3%) of pathogens were multidrug resistant. The death rate among device-associated healthcare infections was 69.2% with a crude excess mortality rate of 37.7%.

Conclusion: The study sheds light on the proportion, types of device-associated healthcare infections, and underlying etiological agents associated with these infections in our institute's ICUs, thereby facilitating a better understanding of the healthcare-associated infection landscape within our facility. Moreover, the susceptibility pattern of pathogens associated with these infections offers crucial information for guiding the selection of appropriate antimicrobial therapies and infection control measures.

## Introduction

Healthcare-associated infections (HAIs) are infections that occur while receiving health care in any setting across the health system, that first appear 48 hours or more after hospital admission, or within 30 days after having received health care and also include all occupational infections, which were not present or incubating at the time of admission/procedure/surgery/using healthcare device [1]. HAIs rank as the third most common adverse event that occurs during the delivery of healthcare services [2]. HAIs create a substantial strain on healthcare systems, resulting in increased rates of sickness, mortality, extended hospital stays, financial expenditures, and emotional and psychological impacts that extend to patients, their families, and communities. Understandably, HAIs are a matter of global concern, drawing attention from governments and patients due to their far-reaching impact on healthcare and public health [3]. 

Individuals admitted to the intensive care units (ICUs) are often in critical condition, with compromised immune systems, and they may have multiple invasive medical devices in use. They also receive prolonged courses of antibiotic treatment and experience extended hospital stays. These factors collectively heighten their vulnerability to HAIs. Every year 0.5 million episodes of HAIs are being diagnosed in ICUs [1].

Based on data from the World Health Organization (WHO), as presented in the report “The Global Impact of Healthcare-Associated Infections” spanning from 1995 to 2008, the occurrence of HAI in developed nations varies from 5.1% to 11.6%. Nonetheless, the impact of HAI is significantly greater in developing countries, particularly among high-risk populations such as those admitted to ICUs [4].

In India, the occurrence of these infections in ICUs is notably elevated, with rates ranging from 9.6% to 17.7%. [5,6] According to a study examining device-associated infections conducted from 2003 to 2008 in 173 ICUs across 25 countries in Africa, Asia, Europe, and Latin America, the additional mortality rates among adult patients for catheter-related urinary tract infections, bloodstream infections (BSIs), and ventilator-associated pneumonia (VAP) were 18.5%, 23.6%, and 29.3%, respectively [7].

In modern healthcare, invasive procedures, surgeries, indwelling medical devices, and prosthetic implants are associated with HAIs and these infections are broadly classified into device-associated and non-device-associated infections. Device-associated healthcare infections such as central-line-associated bloodstream infections (CLABSI), catheter-associated urinary tract infections (CAUTI), and VAP constitute the majority of HAIs in ICUs [1].

Most of the device-associated healthcare infections are caused by antibiotic-resistant microorganisms which pose a serious threat to patients' and healthcare workers' safety. The inaugural worldwide report on infection prevention and control, published by the WHO in 2022, highlighted that antimicrobial resistance (AMR) has emerged as a primary global cause of mortality. Among the prominent AMR pathogens responsible for this significant burden, five out of six are predominantly linked to healthcare-associated sources. Around 75% of disability-adjusted life years (DALYs) attributable to AMR in European Union and European Economic Area countries are a result of HAIs [3]. Hence, it is crucial to know the burden of device-associated healthcare infections and the antibiotic susceptibility pattern of pathogens associated with these infections as rates of device-associated healthcare infections vary in different hospitals or the same hospital at different periods [8]. It also aids in formulating coherent infection prevention and control activities to help reduce device-associated healthcare infections, the spread of multidrug resistant (MDR) organisms, and appropriate treatment of these infections. Hence, this study was undertaken to examine and establish the baseline rates of HAIs linked to medical devices in two adult multidisciplinary ICUs within a tertiary care institute.

## Materials and methods

Study design and study setting

This hospital-based prospective observational study was conducted at the two adult multidisciplinary ICUs of a 750-bed tertiary care institute in Central India over nine months from August 2022 to April 2023 with approval from the Institutional Ethics Committee (UH/IEC/2022/474).

Study participants

All patients 18 years and above admitted to the ICUs for a minimum of two calendar days were included in the study. Patients who were admitted to both ICUs with different medical issues and symptoms, and later exhibited signs of infection that were not related to their initial diagnosis upon admission, were considered eligible for participation in the study. Patients showing evidence of existing infections on admission or initial culture taken within 48 hours of admission yielding microorganisms were excluded. Patients who were moved out of the ICUs within the first 48 hours of their admission, pregnant patients, and patients with surgical site infections were also excluded from the study's participant group.

Data collection

Targeted surveillance for device-associated healthcare infections (CAUTI, CLABSI, VAE) was conducted in both ICUs by daily rounds. Patients were under surveillance for device-associated healthcare infections until they were either discharged from the ICU or succumbed to their condition. Members of the infection control team were involved in the daily monitoring of ICUs and recording data on specifically designed surveillance forms as per Centre for Disease Control National Health Survey Network (CDC-NHSN) criteria to identify device-associated healthcare infections.

Patient details including date of admission, demographic profile, diagnosis, underlying comorbidities, date and site of device insertion, presence of symptoms, laboratory investigations, culture reports including isolated pathogens, antibiogram results, and patient outcomes were recorded.

Definitions

VAE, CLABSI, and CAUTI were defined as per the CDC/NHSN 2016 surveillance criteria for HAI and criteria for specific types of infections in the acute care setting [9].

**Table 1:** CDC/NHSN 2016 surveillance criteria for healthcare-associated infection

DA-HAI	Surveillance Criteria
VAE	The VAE was detected by using a combination of specific criteria, which included a decline in respiratory condition after a period of stability or improvement on the ventilator (such as a significant increase in the minimum fraction of inspired oxygen by at least 20 points or a positive end-expiratory pressure increase of at least 3 cm of water during the baseline period). Additionally, indications of infection or inflammation were considered, such as a body temperature exceeding 38°C or dropping below 36°C, a white blood cell count of 14,000 cells/mm3 or lower, and the initiation of a new antimicrobial medication that was continued for at least 4 calendar days. Furthermore, laboratory evidence of a respiratory infection, such as the presence of purulent respiratory secretions observed under a microscope or the identification of a positive culture in respiratory specimens, was taken into account. VAE algorithm was only applied to patients who were both mechanically ventilated and at least 18 years old.
CLABSI	This pertains to a bloodstream infection that was verified by laboratory tests. To confirm it, there needed to be either one positive blood culture for a known pathogen or two or more positive blood cultures for common commensal organisms. Furthermore, the patient had to exhibit at least one of the following symptoms: fever, low blood pressure, or a slow heart rate. It's essential to emphasize that this infection should not have originated from any other body site, and the central line, a catheter used for intravenous access, must have been in position for a minimum of two consecutive days.
CAUTI	It was recognized when a patient displays signs of a urinary tract infection, such as fever, urgency, increased urination, painful urination, tenderness in the lower abdomen, or back. Furthermore, a urine culture should indicate the presence of a minimum of 10^5^ colony-forming units per millilitre of urine, with no more than two different types of microorganisms. This diagnosis applies when the patient has had an indwelling urinary catheter for more than two consecutive days.

Microbiological sampling and culture

Different samples such as catheterized urine, endotracheal secretions, tracheal aspirates, and peripheral and central line blood cultures from suspected cases of respective device-associated healthcare infections were collected aseptically by the trained nursing staff of ICU and immediately submitted to the microbiology laboratory for Gram’s stain, culture and sensitivity. Cultures were done using 5% Blood agar, and MacConkey’s agar and plates were incubated at 37°C for 18-24 h. Isolated pathogens were identified by standard laboratory protocol including examination of colony morphology, Gram stain, and biochemical tests. Antibiotic susceptibility testing was performed using a Vitek-2 compact (BioMérieux, France) automated system to determine Minimal Inhibitory Concentrations (MIC). Non-repeat-positive cultures with antibiogram were included in the study. Different types of device-associated healthcare infection rates and device utilization ratios (DURs) were calculated using the following formulas [9].

Patient Days

Total number of days that all patients were in the ICU during the selected period

Device Days

Total number of days of exposure to the device (ventilator, central line, or urinary catheter) by all patients in the selected population during the selected time period.

Device Utilization Ratio

Dividing the total number of device days by the total number of patient days. 

Rates of Device-Associated Healthcare Infection

Episodes of specific device-associated infections (VAE/CLABSI/CAUTI) divided by respective device days multiplied by 1,000; to be expressed in terms of rates per 1,000 device days.

Crude Mortality Rate

It is the total number of deaths to total number of patients.

The statistical analysis involved the input of data into Microsoft Excel, followed by analysis using Stata version 14.0. Categorical variables were presented as proportions, while outcomes such as mortality were represented as rates along with their corresponding 95% confidence intervals.

## Results

During the study, a total of 1,270 patients were admitted to the ICUs. A total of 5,773 patient days, 1,568 mechanical ventilator days, 1,935 central line, and 3,936 urinary catheter days were studied. Twenty-eight episodes of device-associated healthcare infections were detected in 26 patients, indicating an overall infection rate of 2.04% or an overall incidence of five device-associated healthcare infections per 1,000 patient days or 3.76 device-associated healthcare infections per 1,000 device days. The highest HAI rates were observed for ventilator-associated events (8.92 per 1,000 mechanical ventilator days (95% CI: 4.881-14.98) followed by central line-associated BSIs (5.68 per 1,000 central line days; 95% CI:2.84-10.17) (Table 2).

**Table 2:** Distribution of device-associated healthcare infections and device utilization ratio (DUR)

Type of device-associated healthcare infection	Total number of events	Device-associated healthcare infection rate (per 1,000 device days)	Device utilization ratio (DUR)
Central line-associated blood stream infection (CLABSI)	11	5.68	0.33
Catheter-associated urinary tract infection (CAUTI)	03	0.76	0.68
Ventilator-associated events (VAE)	14	8.92	0.27

Device utilization ratios (DUR) for central line (CL), mechanical ventilation (MV), and urinary catheter (UC) were 0.33, 0.27, and 0.68, respectively. Of the 26 patients with device-associated healthcare infections, 14 patients were in the age group of 51-80 years (Table 3). The majority of device-associated healthcare infections, specifically 17 cases, accounting for 65.38%, were documented in male patients. Among the male patients, 35.29% experienced recovery, while 64.71% did not survive.

**Table 3:** Demographic distribution of device-associated healthcare infections

Age	Frequency
Less than 50 years	12 (46.15%)
50 years and above	14 (53.85%)
Total	26

Among the patients admitted to the ICU, the death rate among device-associated healthcare infections (69.2%; 95% CI: 47.6-84.1) was significantly higher (p <0.01) than those without device-associated healthcare infections (30.7%). The crude excess mortality was estimated at 37.7% (95% CI: 20.2%-55.1%). Table 4 shows the clinical outcome of patients who were detected with device-associated healthcare infections.

**Table 4:** Clinical outcome of device-associated healthcare infection cases

Sex	Recovered	Death	Total
Female	02 (22.22%)	07 (77.78%)	09
Male	06 (35.29%)	11 (64.71%)	17
Total	08 (30.77%)	18 (69.23%)	26

Among the 18 cases of device-associated healthcare infections resulting in death, more than three-fourths were (78.6%; N=11) above 50 years of age. We did not find a significant association between age and risk of death due to device-associated healthcare infections. 

Prevalent underlying co-morbid conditions were hypertension (26.9%; N=7) and diabetes mellitus (19.2%; N=5). We found the presence of co-morbidities increased the risk of death among device-associated healthcare infections, but it did not amount to statistical significance. Among hypertensive patients with device-associated healthcare infections, the risk of death was 85.7% compared to 63.1% among those without it. Similar findings were observed in diabetic patients (80%) when compared to non-diabetic patients (66.7%).

Pathogen profile and AMR pattern

**Figure 1:**
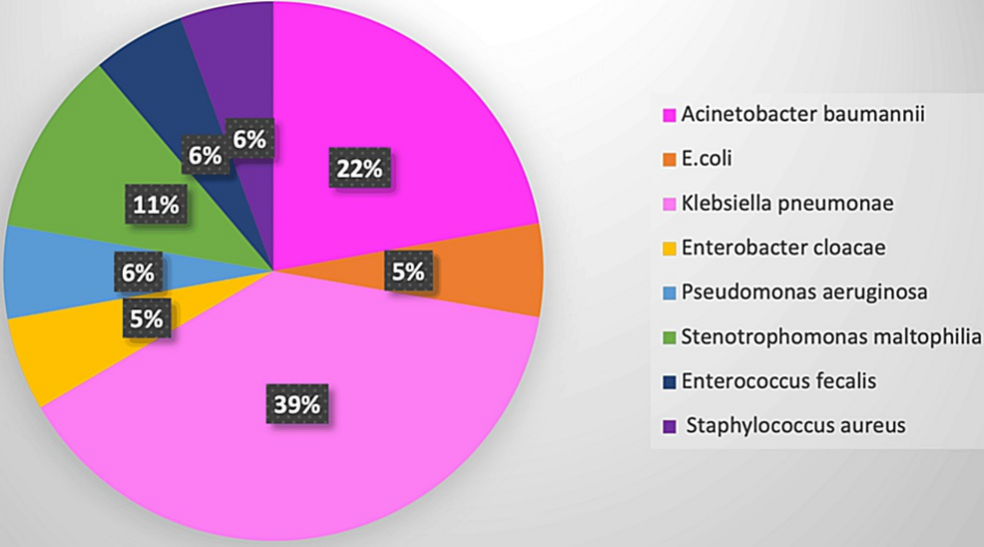
Bacteriological profile of device-associated healthcare infections

Pathogens obtained from device-associated healthcare infections were predominantly Gram-negative bacilli (89%) comprising *Klebsiella pneumoniae* (39%), *Acinetobacter baumanii* (22.2%), *Stenotrophomonas maltophilia* (11%), and *Pseudomonas aeruginosa *(6%). Gram-positive cocci were detected in two (11%) cases only (Figure 1).

A total of 17 pathogens were obtained from device-associated healthcare infections of which most of the pathogens (82.3%) were MDR. Among the Gram-negative pathogens highest resistance was observed for ampicillin (100%), amoxicillin-clavulanic acid (100%), ceftriaxone (92%), ceftazidime (87%), cefepime (79%), meropenem (77%), piperacillin-tazobactam (71%) and cotrimoxazole (79%). Five (71%) isolates of *K*.* pneumoniae* and four (100%) isolates of *Acinetobacter baumannii* were MDR with susceptibility to only tigecycline with MIC of 0.5 µg/mL and colistin with MIC range of 0.5-2 µg/mL. One isolate each of methicillin-resistant *Staphylococcus aureus* (MRSA) and *Enterococcus faecium* was obtained from cases of CLABSI and CAUTI, respectively, with vancomycin MIC of 2 µg/mL for both the isolates. 

## Discussion

Within the ever-evolving landscape of healthcare, vigilance in monitoring and preventing infections, especially those linked to medical devices, is crucial within the ICUs. Given the ICU's pivotal role in patient care, the need for a strong system dedicated to surveillance and prevention of infections associated with medical devices becomes even more pronounced. This study presents the findings of an ongoing active surveillance study on device-associated healthcare infections actively conducted in ICUs of a tertiary care institute. The study observed an overall incidence of device-associated healthcare infections at 3.7 per 1,000 device days or five per 1,000 patient days. Notably, these rates were found to be comparable to those reported in ICU settings in South India [10] and lower than the rates documented by the International Nosocomial Infection Control Consortium (INICC)[11]. In contrast, a study from North India [12] reported substantially higher rates of device-associated healthcare infections in their ICUs, reaching 49.38 infections per 1,000 ICU days.

The lower incidence of device-associated healthcare infections in our institute signifies a commitment to continuous quality improvement initiatives. Since the establishment of our institute, infection prevention measures such as hand hygiene and its audit, the proper utilization of personal protective equipment, barrier nursing, and the implementation of care bundles, have been prioritized. This emphasis on infection prevention measures from the inception of our institute likely contributes to the observed lower rates of device-associated healthcare infections, reflecting a proactive and dedicated approach to ensuring patient safety and quality of care.

Prevalent underlying co-morbid conditions observed in infected patients were hypertension and diabetes mellitus. In a comparable study conducted in Karnataka, India, a notable proportion of patients exhibited conditions like diabetes mellitus and hypertension, along with related behaviors such as chronic smoking [13]. However, comparative prospective studies involving a control group of uninfected patients are required to understand the impact of these factors. 

In this study, the mortality rate among patients with device-associated healthcare infections (69.2%) was markedly higher and statistically significant (p < 0.01) compared to those without such infections. Similarly, a study in Karnataka, India, involving six ICUs at a tertiary care center revealed that the mortality rate among infected patients was nearly twice as high as that of ICU patients without infections [13]. Conversely, a study from Maharashtra, India, indicated that there was no observed increase in mortality among patients with ICU-related infections [5]. The observed excess mortality rate among patients who contracted device-associated healthcare infections (37.7%) was four times greater than the figure reported in the INICC in Lebanon (INICC-2012) (9.8%) [14]. The elevated mortality may be attributed to various factors, including underlying conditions, comorbidities, admission causes, age, criticality, and others, apart from infections. It's noteworthy that we did not evaluate the APACHE IV score and acute physiology score (APS) of patients at the time of admission, both of which are predictors of ICU mortality.

Device utilization ratios were found to be 0.33, 0.27, and 0.68 for central lines, mechanical ventilation, and urinary catheters, respectively. Notably, the DUR for central lines and mechanical ventilators in this investigation were markedly lower than those reported by the INICC for the periods 2007-2012 (0.54 and 0.36, respectively) [11] and 2004-2009 (0.52 and 0.39, respectively) [15], as well as comparable to the figures documented by the INICC in India [16] (0.39 and 0.22, respectively), and in various other studies [12,17]. The risk of device-associated healthcare infections increases with the number of days of device utilization and also device may act as a nidus for colonization by multidrug-resistant organisms. Device utilization ratio can serve as an important component of surveillance for HAIs. Maintaining a balance between efficient device utilization and infection control measures is crucial to reducing the risk of device-associated healthcare infection and MDRO colonization thereby providing safe patient care [17].

The most common device-associated healthcare infection encountered was VAE followed by CLABSI and CAUTI. Our CLABSI and VAE rates were comparable to those reported by INICC, India, and INICC [16,18] (9.4 VAPs, 5.10 CLABSI and 14.1 VAPs, 5.05 CLABSIs, per 1,000 device-days respectively) and much higher than those reported from the US NHSN (1.6 VAPs and 1.1 CLABSIs per 1,000 device-days) [19,20]. Much higher rates of CLABSI were reported from India by Bammigatti et al. from Pondicherry and Patil et al. from Maharashtra (72.56 and 47.31 per 1,000 central line days, respectively) [21,22].

CAUTI rates revealed a wide variation across diverse geographical regions ranging from 1.9 to 34.2 cases per 1,000 catheter days [23,24]. Our CAUTI rate (0.76 per 1,000 urinary catheter days) was lower than the NNIS rate of 3.3 per 1,000 catheter days and the overall INICC CAUTI rates [25,26] Conforming to care bundles for the insertion and maintenance of catheters accompanied by providing prompt feedback to healthcare workers and sharing HAI data with hospital administration and ICU staff, could potentially result in a decrease in the observed rates of CAUTI as suggested by findings in another study [10].

The predominance of Gram-negative infections (*K. pneumoniae* (39%), *A. baumannii* (22%), *Stenotrophomonas maltophilia* (11%), and *P. aeruginosa* (6%) was observed which was reported by several other surveillance studies in India [5,6,10]. Gram-negative bacilli represented the most common nosocomial isolates in many studies from low and middle-income countries [8]. Pathogens isolated in the present study were predominantly MDR. Inappropriate antibiotic usage could result in an increased occurrence of MDR organisms, thereby necessitating more frequent utilization of reserved medications such as colistin and tigecycline by clinicians. Additional studies conducted in India have presented data on the frequency of HAIs caused by pathogens exhibiting AMR phenotypes [10,27]. Consequently, it is of utmost importance to prioritize the implementation of antimicrobial stewardship programs to ensure the best possible utilization of antibiotics. Further studies are needed to understand the relationship between device-associated healthcare infection rates and MDR organisms and to develop more effective prevention and control strategies.

Limitations

The study findings were confined to a short timeframe and exclusive to two adult multidisciplinary ICUs within a single tertiary care institute. These findings may not be universally applicable to diverse public or private hospital settings. The lack of information on the severity of illness at admission constrained our ability to include these variables when identifying risk factors for device-associated associated healthcare-infections. Despite these limitations, the study furnishes clinicians with valuable insights into the prevalence of device-associated healthcare infections, particularly given the limited availability of such data in published literature from Central India.

## Conclusions

This study provides a foundational understanding of device-associated healthcare infections within the ICUs of our institution. The observed rate of these infections in ICUs was lower than the rates reported in many hospitals in developing nations. Ventilator-associated events were found to be the most prevalent, followed by central line-associated BSIs. Notably, *K. pneumoniae *and *A. baumannii *were frequently isolated, and a significant prevalence of drug resistance was observed among these pathogens. The study highlighted the substantial impact of these infections on ICU mortality. Surveillance and reporting of such infections play a pivotal role in tertiary care institutes, helping to identify areas for improvement in infection control protocols and resource allocation. Furthermore, it is instrumental for comparing infection rates and practices with peer institutions, fostering collaborative learning, and identifying best practices that contribute to enhanced patient outcomes and policymaking.
